# Homomeric and Heteromeric α7 Nicotinic Acetylcholine Receptors in Health and Some Central Nervous System Diseases

**DOI:** 10.3390/membranes11090664

**Published:** 2021-08-29

**Authors:** Virginia Borroni, Francisco J. Barrantes

**Affiliations:** 1 Instituto de Tecnología en Polímeros y Nanotecnología (ITPN-UBA-CONICET), Universidad de Buenos Aires, Ciudad Universitaria, Buenos Aires C1127AAR, Argentina; mvirborroni@gmail.com; 2 Laboratory of Molecular Neurobiology, Institute for Biomedical Research, UCA–CONICET, Faculty of Medical Sciences, Catholic University of Argentina, Av. Alicia Moreau de Justo 1600, Buenos Aires C1107AAZ, Argentina

**Keywords:** cholinergic neurotransmission, neurotransmitter receptor protein, nicotinic, acetylcholine receptor, α7 nAChR, dendritic spine, synaptopathy

## Abstract

Nicotinic acetylcholine receptors (nAChRs) are pentameric ligand-gated ion channels involved in the modulation of essential brain functions such as memory, learning, and attention. Homomeric α7 nAChR, formed exclusively by five identical α7 subunits, is involved in rapid synaptic transmission, whereas the heteromeric oligomers composed of α7 in combination with β subunits display metabotropic properties and operate in slower time frames. At the cellular level, the activation of nAChRs allows the entry of Na^+^ and Ca^2+^; the two cations depolarize the membrane and trigger diverse cellular signals, depending on the type of nAChR pentamer and neurons involved, the location of the intervening cells, and the networks of which these neuronal cells form part. These features make the α7 nAChR a central player in neurotransmission, metabolically associated Ca^2+^-mediated signaling, and modulation of diverse fundamental processes operated by other neurotransmitters in the brain. Due to its ubiquitous distribution and the multiple functions it displays in the brain, the α7 nAChR is associated with a variety of neurological and neuropsychiatric disorders whose exact etiopathogenic mechanisms are still elusive.

## 1. Introduction

Brain nicotinic acetylcholine receptors (nAChRs) have been classically associated with excitatory neurotransmission [1] and its modulation (see ref. [2]), but these are only fragmentary facets of their functional palette; their cellular distribution in development and adulthood [3,4] and subcellular localization (presynaptic, but also perisynaptic and postsynaptic [5]; reviewed in [6]) add complexity to these apparently primary roles. The activation of nAChRs mediates fast synaptic transmission in certain brain areas and in autonomous ganglionic neurons, but most nAChRs in the brain are located at presynaptic sites. Thus, nAChRs exert mostly neuromodulatory roles (see review in Picciotto and coworkers [7]) on neurotransmitters and their receptors, e.g., modulating the release of other neurotransmitter [8,9] or of its own neurotransmitter, acetylcholine (ACh) [10,11,12]. In addition to these modulatory roles, exerted most importantly on cerebral cortex circuits, hippocampus, basal ganglia and cerebellum, nAChRs regulate synapse development [13], cell viability [14], neurite outgrowth [15] brain energetic homeostasis [16], and the establishment of functional networks during neural development ([17] and see review in [18]).

Due to their strategic location and enrichment in areas involved in working memory acquisition, maintenance, and retrieval, nAChRs in the hippocampus and cerebral cortex play major roles in cognition and mnemonic functions [19,20] and modulate prefrontal cortex functions involved in consciousness [21]. At present, we probably know only a fraction of the roles played by nAChR subtypes in the functioning of the human brain [22]. Differences in regional and cellular localization allow the cholinergic system to control a variety of functions using a simple molecule, such as ACh, as a trigger. The multiplicity of important roles played by nAChRs means that their dysfunction is related to various disease conditions, some of which are beyond their involvement in classic neurotransmission (see “volume transmission” below). The association of nAChRs with brain pathologies, such as glioblastoma and neuroAIDS, is increasingly being documented [23,24]. 

Owing to historical facts related to its isolation and early biochemical characterization, the nAChR became the paradigm member of the pentameric ligand-gated ion channels (pLGIC), a superfamily of structurally related neurotransmitter receptors that include both the nicotinic-type neuronal and muscle-type nAChRs, γ-amino butyric acid (GABA)_A_ receptors, the glycine receptor, and the 5-HT3 subtype of serotonin receptors. nAChRs are formed by the combination of five subunits organized pseudo-symmetrically around a central pore. The binding of the natural agonist, ACh, triggers the opening of the pore, the ensuing entry of cations into the cell, and the resultant depolarization of the membrane. The sustained presence of the agonist at the agonist-binding site leads the nAChR to a desensitized state in which the receptor is closed but the agonist remains bound. In mammals, 17 genes that code for nAChR subunits have been identified, characterized, and their genes cloned. Unlike the muscle type nAChR, which is formed by the combination of α, β, δ, and γ (or ε) subunits, nAChRs in the central nervous system (CNS) comprise a combination of different α and β subunits or even α subunits alone. The homomeric α7 and α9 or heteromeric α2–α7, combined with β2, β3 and β4 and α9/α10 heteromers, nAChR subtypes result from the grouping of these subunits into pentameric oligomers of ca. 270,000 Da [6,25]. The association of α7 subunits with the subunits present in neuronal receptors that do not bind α-bungarotoxin, such as the α5 [26], β2 [27], β3 [28], and β4 subunits [29], results in functional heteromeric ion channels.

In terms of abundance, the predominant nAChR subtype expressed in the brain is the heteromeric α4β2-subtype, followed by the homomeric α7 nAChRs [6]. The latter are, in fact, homomeric in rats (i.e., containing only α7 subunits [30,31], but may be homomeric α7, α8, or α7/α8 subunit-containing nAChRs in chicks [32,33]). The antagonist α-bungarotoxin binds with high affinity to the α7 nAChR. The α7 nAChR is more broadly distributed than other neurotransmitters receptors and is expressed in neurons (pre, post and perisynaptically), microglia, astrocytes, autonomic ganglia, pulmonary epithelial cells (with an important role in some lung carcinomas), lymphoid cells, and endothelial cells. It is highly permeable to Ca^2+^ and also stimulates Ca^2+^ release from intracellular stores; its activation can, therefore, be coupled to multiple Ca^2+^-dependent signaling systems that employ intracellular second-messengers and non-classical (“volume”) graded transmission, in which ACh release sites are distant from their receptor target [34,35,36,37,38]. Another characteristic property of this receptor is its fast desensitization kinetics.

Affinity purification using the α7-selective antagonist α-bungarotoxin led Gotti and coworkers to identify and biochemically characterize a heteromeric α7β2 subtype of neuronal receptor in the basal forebrain of humans and WT mice [36]. Ligand stimulation of α7 nAChRs regulates neurite outgrowth through G-protein coupled receptor (GPCR) pathways [37]. A direct interaction between a G-protein and a G-protein binding motif in the intracellular domain of the α7 nAChR was demonstrated by the group of Kabbani [38]. The heteromeric α7β2 nAChR in rat basal forebrain cholinergic neurons has different biophysical and pharmacological properties than those of the homomeric α7 nAChRs present in ventral tegmental area neurons. The α7β2 nAChR exhibits slower rise and decay kinetics than the homomeric α7 nAChR [39]. Moreover, a reduced potency for nicotine and significantly less activity of type I PAMs has been reported for heteromeric receptors [36,39,40]. The α7β2 heteromeric subtype is highly sensitive to functional inhibition by oligomeric amyloid peptide Aβ1-42, an observation that acquires particular relevance in the context of Alzheimer disease [33]. Indeed, a recent study showed that oligomeric Aβ1-42 alters the excitability of a specific group of neurons in the basal forebrain by increasing heteromeric α7β2 nAChR open-dwell times. Additionally, oligomeric Aβ1-42-α7β2 nAChR interaction reduces the acquisition and retention of spatial reference memory in the APP/PS1 mouse model of AD [41]. These observations are suggestive of heteromeric α7 involvement in the early cognitive deficits present in AD. It should be noted that most studies conducted on tissues where α7 and α7β2 nAChR are co-expressed, e.g., the basal forebrain, are not able to dissect the contribution of each subtype of receptors. This is because the ligands used to assay the homomeric receptors also bind heteromeric receptors [42]. Many conclusions about the role of homomeric α7 nAChRs should be revisited to consider the contribution of heteromeric α7β2 nAChR.

**Figure 1:**
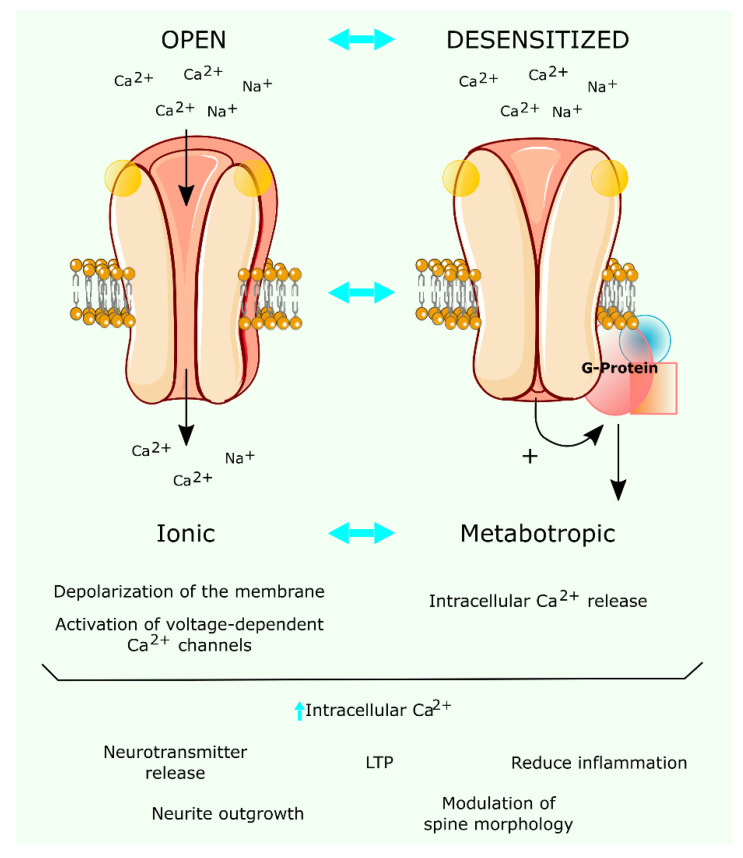
Ionotropic and metabotropic signaling of α7 nAChRs. Ionotropic signaling (left flow chart): upon ligand binding, the homomeric α7 nAChR channel opens, allowing the flux of Ca^2+^ and Na^+^ into the target cell, which produces the depolarization of the membrane. This triggers the opening of voltage-activated Ca^2+^ channels and the intracellular Ca^2+^ concentration increases further. Metabotropic signaling (right flow chart): sustained exposure to the agonist shifts the α7 nAChR conformation to the desensitized state, under which ACh or exogenous agonist molecules are bound to the oligomer, but the channel remains closed, and no ion permeation occurs. However, desensitized α7 nAChRs can couple to G-proteins that activate a cascade of signals ending in the release of Ca^2+^ from intracellular stores (e.g., the endoplasmic reticulum). These signaling cascades may have different effects depending on the nature of the stimulus.

The α7 nAChR operates not only as a rapid ligand-gated ion-channel but also as a metabotropic receptor protein (Figure 1). The α9-subunit-containing nAChR have also been shown to display metabotropic functions. Homomeric α9 nAChR or heteromeric α9-α10 nAChR heterologously expressed in *Xenopus* oocytes do not generate ion currents when stimulated with phosphocholine, in contrast to what is observed with choline, a metabolite of ACh [43] that also acts as an agonist of the α7 nAChR [44]. Phosphocholine, the precursor of phosphatidylcholine, inhibits the ATP-dependent release of the pro-inflammatory cytokine interleukin-1β from human and murine monocytes via a mechanism involving α9-α10 nAChRs [45]. It is unlikely that these two modes of action work as separate mechanisms in vivo due to the fast desensitization rate induced by the endogenous neurotransmitter ACh. However, separation of the two signaling pathways can be accomplished by human-tailored pharmacological intervention (see below). These dual functional properties endow the α7-type of nAChRs with interesting possibilities for pharmacological interventions in a wide range of disease conditions associated with this receptor. The α7 nAChR can also be activated by choline, the precursor metabolite of the endogenous neurotransmitter in the presynapse and the degradation product of ACh generated in the synaptic cleft by the action of the enzyme acetylcholinesterase [44]. Choline is a selective full agonist for α7 nAChR at physiological concentrations [46]. The generation of choline upon ACh hydrolysis could provide a mechanism to extend signaling selectively through the α7 nAChR [1]. The subsequent uptake of choline by the high-affinity choline uptake mechanism spatiotemporally localizes choline near the cholinergic terminals [39]. However, the timing for choline clearance is long enough (5–10 s) to allow diffusion of this metabolite outside the synaptic region [47]. The presence of choline beyond the synaptic area can activate extrasynaptic receptors in neurons or glial cells [1]. The contribution of choline to the entire cholinergic response is not always appreciated; it may bear relevance to the therapeutic use of cholinesterase inhibitors, as is the case in Alzheimer disease. 

A549 adenocarcinoma, U87MG human grade-IV astrocytoma, and GBM5 glioblastoma cells lines express α7 and α9/α10 nAChRs subunits [23,48]. The application of agonists such as choline or nicotine activates Akt/ERK anti-apoptotic signals and promotes cell proliferation [23,42]. The doses used to obtain responses are below those required to record currents in electrophysiological experiments, suggesting that metabotropic signaling is involved in the former case. An antiglioblastoma agent was synthesized based on the structure of a peptide displaying strong anti-α7 nAChR antagonism [49]. Metabotropic signaling is usually observed after ligand binding to α7 nAChRs in many non-neuronal cells, such as immune cells, where α7 nAChRs modulate the release of cytokines. The role of α7 nAChRs in HIV-associated neurocognitive disorders (HAND) was recently demonstrated by Zhao and coworkers [24]. α7 nAChR activation improved locomotor activity, learning, and memory deficits in an inducible model of HAND that expresses the HIV soluble protein Tat. Tat increased α7 nAChR expression in microglia and astrocytes but decreased α7 nAChR expression in neurons, and this ratio was reversed upon treatment with the α7 nAChR positive allosteric modulator (PAM) PNU-125096 (see below). PSD95, a major scaffolding protein with an important role in synapse formation, increased its expression in the hippocampus and cortex upon PAM treatment, suggesting that its recovery is a compensatory mechanism coincident with the observed improvements in cognitive, mnemonic, and locomotive functions [24]. 

## 2. Dendritic Spines, the Postsynaptic Specialization of Excitatory Synapses in the Brain

Chemical synapses in the CNS have an overall similar architecture: a presynaptic region separated by a 15–20 nm gap (the synaptic cleft) from the postsynaptic region at the receiving end [50] (for a recent review see [51]). This structural design essentially responds to a key functional requisite: the vectorial transmission of the chemical signal. The presynapse is usually an en passant axon and often establishes multiple contacts with dendrites or the soma of the target neuron, thus forming axo-dendritic or axo-somatic synapses, respectively, but it can also involve another axon, in which case the synapse is of the axo-axonic type. The most typical postsynaptic partner is a dendrite, classically associated with excitatory glutamatergic neurotransmission, whereas inhibitory synapses are more frequently found on neuronal soma or dendritic shafts, and less frequently on axons. The multiple combinations of pre- and postsynaptic components in diverse brain regions and layers of the cerebral cortex give rise to a wide range of synaptic morphologies. This structural diversity is expanded by the chemical diversity of the neurotransmitter systems involved, which confers specificity to the synapse, since, usually, only one neurotransmitter operates as the main signaling system in a given synapse (although there are a few exceptions to this rule). 

Excitatory synapses in the brain operate via glutamate receptors (*N*-methyl-*D*-aspartate (NMDA) receptors), α-amino-3-hydroxyl-5methyl-4-isoxazole-propionate (AMPA) receptors, and kainate receptors. These three subtypes of excitatory neurotransmitter receptors belong to a superfamily of tetrameric proteins, which normally reside in a postsynaptic specialization termed dendritic spine. In their fully developed form, dendritic spines are small mushroom-shaped protrusions. Dendritic spines have a head volume between 0.005 and 1 μm^3^ and a neck between 0.1–0.5 μm of diameter and 0.1–2.5 μm length. Having minute volumes in the order of femtoliters, they nevertheless harbor a wide variety of receptor proteins, scaffolding proteins, synaptic adhesion molecules, and enzymes involved in the local biosynthesis and catabolism of proteins and lipids of the postsynapse. Some nAChRs also occur in dendritic spines. A key localization of α7 nAChRs in the hippocampus is that established on the perisynaptic annulus of CA1 pyramidal cell dendritic spines [52]. 

**Figure 2:**
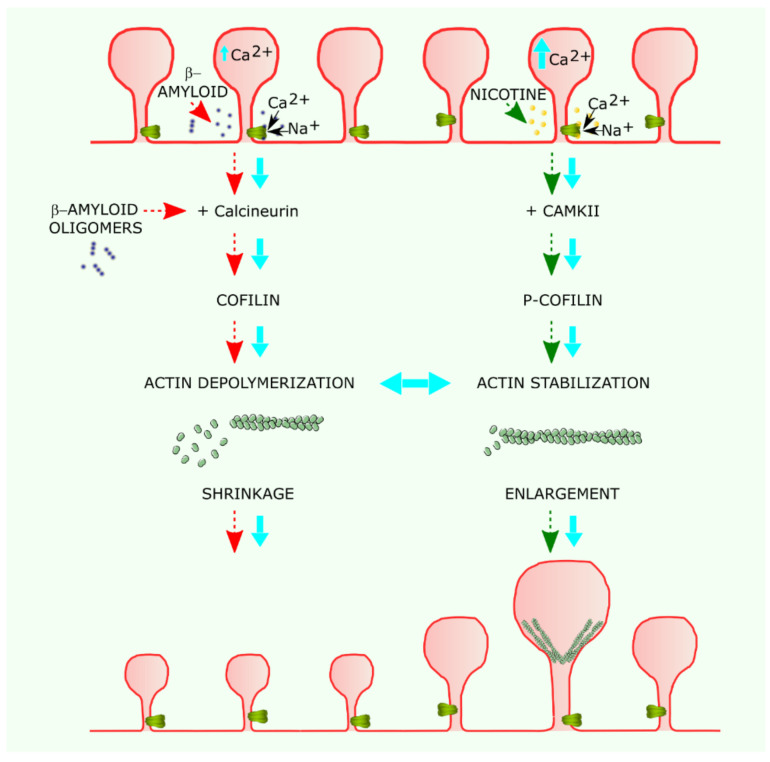
Possible mechanism of α7 nAChR modulation of dendritic spine morphology and its alteration in Alzheimer disease. The vertical flow diagram on the left shows the pathological cascade, in which β-amyloid oligomers activate α7 nAChR and the calcineurin–cofilin pathway. The increase in intracellular calcium concentration reinforces calcineurin activation. Activated calcineurin dephosphorylates cofilin, which, in turn, depolymerizes actin filaments, leading to spine shrinkage and dysmorphism. Diffusion of cofilin to neighboring spines acts like a chain reaction, spreading the shrinkage along the dendrite. The flow diagram on the right illustrates the protective action of nicotine, which activates α7 nAChRs leading to intracellular calcium increases that activate CAMKII, which, in turn, leads to phosphorylated cofilin (p-cofilin), stabilizing actin filaments. The polymerization and stabilization of actin filaments is associated with spine enlargement.

Spines are very dynamic structures that respond to activity by changing their size and shape. These changes are driven and sustained by rearrangements of the actin cytoskeletal meshwork underneath the postsynaptic membrane and are linked to functional modifications that result in synaptic plasticity. Spines can enlarge in response to repetitive stimulation or shrink under low frequency stimulation. Both processes depend on intracellular Ca^2+^ increases. In the case of spine enlargement, when the cation increase is sufficiently high, it activates CaMKII, whereas a moderate increase activates calcineurin, resulting in a shrinkage of the spine (Figure 2). Interestingly, enlargement is confined to the stimulated spine, while shrinkage can spread to adjacent spines along the dendrite. 

Dendritic spines can spontaneously change their overall morphology, independently of synaptic transmission. Indeed, the same profile of synaptic size distribution was observed in neurons where activity was blocked as in neurons with intact activity [53]. This intrinsic dynamic is the combined result of the turnover of scaffold protein molecules, the extracellular matrix, and the interaction of synapses with the glia. Whether this intrinsic spine dynamic is the reflection of the particular genetic background or the starting point from which experiences start to model the individual brain is not clear. However, in mice carrying mutations associated with animal-model schizophrenia spectrum disorders, a loss of small spines is observed, whereas in mice models of fragile X syndrome and autism spectrum disorder, spine turnover rate is accelerated (independently of activity) and the result is an enrichment in small synapses. Moreover, exposure of mouse cultured hippocampal neurons to soluble Aβ oligomers results in a time-dependent enrichment in longer, elongated and dysmorphic spines [54]. 

Small spines are more dynamic than large spines and are thought to store new memories [55,56]. Interestingly, new spines generated after learning are added to preexisting connections [56,57]. This phenomenon is thought to preserve information acquired from changes originated by intrinsic dynamic mechanisms by coding newly acquired information in synapses with multiple spines and connections. 

Application of the cholinergic agonist nicotine has been reported to induce spine enlargement, as assessed using structured illumination super-resolution optical microscopy [58]. Activation of α7 nAChR protects synapses potentiated by theta pattern stimulation in hippocampal slices, while receptor desensitization has the opposite, de-potentiating effect. De-potentiation is mediated by actin depolymerization [59]. α7 nAChRs may thus control dendritic spine shape and strength through modulation of actin dynamics. Indeed, α7 nAChR was shown to directly interact with guanine-exchange factors of Rho and Rac small-G proteins in hippocampal synapses in vitro [37]. Moreover, nicotine was shown to activate CAMKII in hippocampal neurons from mice with depressive-like behavior. The specific α7 nAChRs antagonist mecamylamine blocks this activation [60]. Furthermore, Ca^2+^ increases at levels capable of activating Ca^2+^ signaling systems have been observed upon activation of α7 nAChRs. This increase is the result of direct Ca^2+^ entry through the nAChR ion channel and the consequent depolarization of the membrane and activation of voltage-dependent Ca^2+^ channels followed by the release from Ca^2+^ intracellular stores, including ER, at longer times [61]. These changes in intracellular Ca^2+^ concentration act as a signaling system that controls the remodeling of dendritic spines (Figure 2).

## 3. Fine Structure of the Neuronal nAChR Molecule

Until recently, the peripheral muscle-type nAChR found in the neuromuscular junction of vertebrates and the electroplaque of electric fish constituted the best studied nicotinic receptor [62] and the first source of information on the 3D structure of this family of ion channel proteins. All nicotinic receptors share a common architectural framework: a relatively hydrophilic extracellular amino-terminal moiety that carries the orthosteric agonist-recognition site and is exposed to the synaptic cleft, three hydrophobic transmembrane domains (M1–M3) followed by a large intracellular loop, and a fourth hydrophobic transmembrane domain (M4) [63]. The M2 segment lines the walls of the ion-conducting central pore and key amino acids determine ionic selectivity and channel gating properties of the receptor pentamer. We also know that a sub-family of CNS nAChRs is sensitive to the selective antagonist α-bungarotoxin (the α7, α8, α9, and α10 subunit-containing nAChRs) whereas the rest, the heteromeric nAChRs formed by α/β combinations, are insensitive to this toxin. However, we ignored whether these neuronal homomeric and heteromeric α7 nAChRs had a pentameric structure similar to that of the muscle-type receptor, with five subunits organized pseudo-symmetrically around a central ion-conducting pore. It is only in the last decade that structural studies have focused on the crystal structure of the neuronal type of nAChR. 

**Figure 3:**
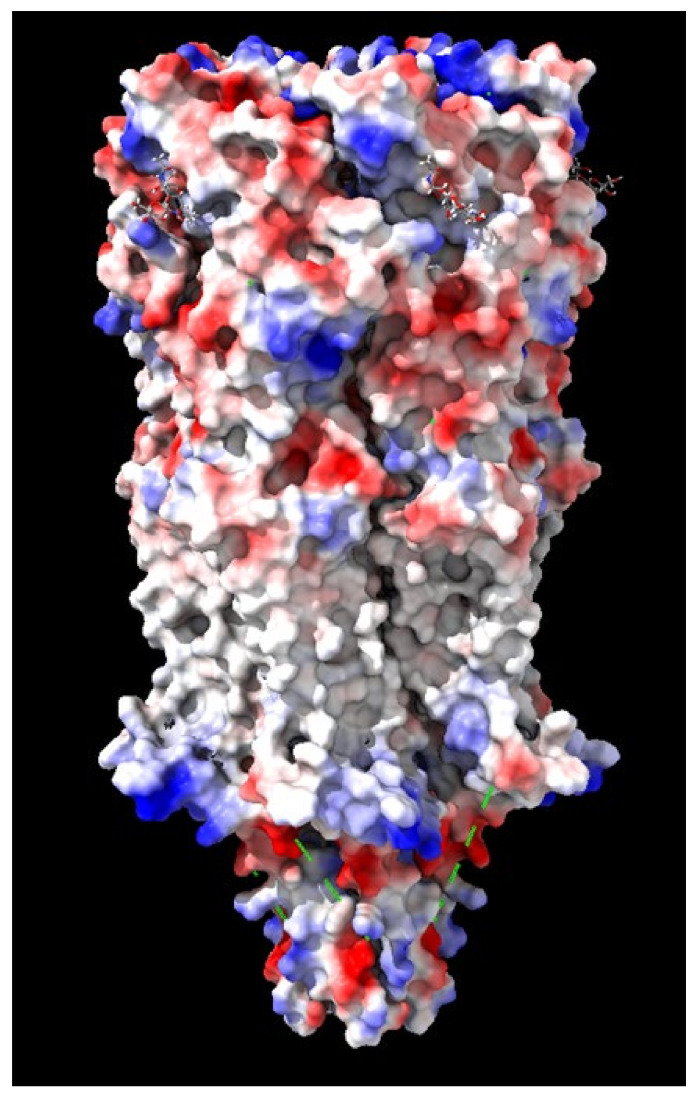
Reconstruction of the single-particle cryo-EM data of the homopentameric α7 nAChR. The structure of the receptor protein resolved at 3.9 Å resolution by Noviello and coworkers [65] is shown in surface rendering. Two epibatidine molecules (ball-and-stick representation, and a third molecule partly seen at the top right) are seen bound to crevices in the extracellular domain of the nAChR. The receptor protein was fused to soluble cytochrome b562 to facilitate crystallization. PDB entry: 7KOQ; EMDataResource: EMD-22980. Image created using the software PyMol Molecular Graphics System vers. 2.4.1. from Schrödinger, LLC, New York, U.S.A.

The most abundant neuronal nAChR, the α4β2 subtype, accounts for 90% of the high affinity nAChR sites in the mammalian brain [63]. Its structure was solved in 2016 in a presumably non-conducting, desensitized conformation in the presence of the agonist nicotine [64]. The X-ray crystallographic structure of this neuronal receptor at 3.94 Å resolution was recently determined using X-ray crystallography and cryo-electron microscopy. The reconstructed structure of the receptor, fused to soluble cytochrome b562, was obtained in various forms, one of them with bound epibatidine, a natural chlorinated alkaloid secreted by the Ecuadorian frog *Epipedobates anthonyi* and poisoned dart frog from the *Ameerega* genus (PDB entry: 7KOQ) (Figure 3). Two other structures determined by this group comprised the α7 nAChR with bound epibatidine and the positive allosteric modulator PNU-120596, which rendered the receptor in an active configuration, and the receptor bound to the quasi-irreversible competitive antagonist α-bungarotoxin, corresponding to the inactive conformation [65]. The neuronal α4β2 nAChR follows the architectural design of the muscle-type receptor, as compared with the cryo-EM reconstruction of electric fish nAChR from *Torpedo* at 4.0 Å resolution [56]. Experimental conditions were optimized to obtain purified human α4β2 receptor with almost exclusively 2α:3β stoichiometry. In a subsequent work, the same group resorted to cryo-electron microscopy and produced a mixture of two α4β2 nAChR isoforms, having the stoichiometries 2α:3β and 3α:2β, respectively, and studied the complex of these receptors with the agonist nicotine [66]. When nicotine is added to cell cultures, as with smoking, the 2α:3β isoform increases in detriment of the other. Conversely, autosomal-dominant nocturnal frontal lobe epilepsy shifts the stoichiometry in favor of the 3α:2β isoform [67,68]. These changes in subunit stoichiometry are associated with thermodynamically distinct conformations and different subunit interface interactions, with consequences on agonist sensitivities [66].

In heteromeric nAChRs, the interface between one α subunit along with the adjacent non-α subunit lodges the agonist binding site. Therefore, there are two binding sites per receptor monomer. These sites were confirmed in the extracellular domain of the α2β2 receptor crystals in X-ray diffraction studies at 3.2 A [69]. In contrast, the α7 nAChR possesses five binding sites at the interfaces of the five homologous subunits. The α7 nAChR can, thus, be activated at lower levels of occupancy, even with only one ACh molecule bound to a single orthosteric site [70]. Higher levels of occupancy lead to desensitization. As previously mentioned, the α7 nAChR has very fast desensitization kinetics as compared to other nAChRs subtypes, a property that is advantageous in the termination of synaptic transmission mediated by these receptors. 

## 4. Functional Pharmacology of Brain nAChR-Mediated Synaptic Plasticity 

Cholinergic neurotransmission plays a particularly relevant role in the cerebral cortex, where it modulates the functional status of pyramidal neurons. Inhibitory impulses mediated by nicotinic receptors on these cells are rapid. Excitatory signaling mediated by ACh on α4β2 nAChRs stimulates the release of glutamate, the main excitatory neurotransmitter in the brain. Activation of α7 nAChRs induces long-term potentiation (LTP) and inhibits induction of long-term depression (LTD), two manifestations of synaptic plasticity. In rodents, the cornu ammonis (CA1) hippocampal neurons play a key role in the processing of hippocampus-dependent memory. In humans, the CA1 neurons have been reported to be critical for the so-called autobiographical memory, autonoetic consciousness, and mental time travel. In patients with acute transient global amnesia, an infrequent nosological entity, MRI detects highly focalized lesions confined to the CA1 hippocampus, an observation that highlights the importance of this region for autobiographical memory retrieval [71].

**Figure 4:**
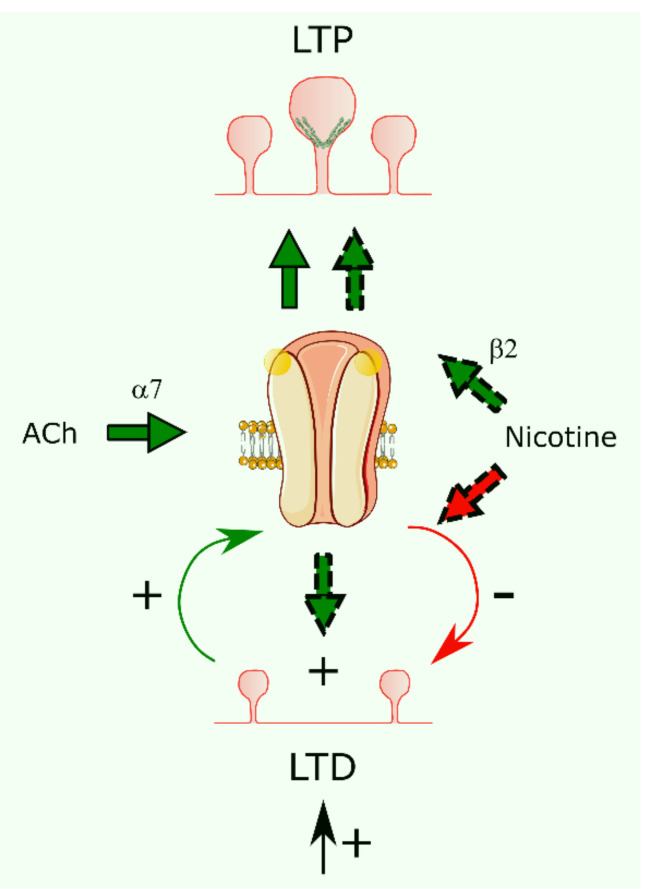
Modulation of synaptic plasticity by cholinergic agonists. Induction of long-term depression (LTD) stimulates α7 nAChR, which, in turn, inhibits LTD (solid line arrows). Nicotine application releases the inhibition and increases LTD (dash line arrows). Long-term potentiation (LTP) can be induced by the endogenous release of ACh through homomeric α7 nAChRs or by nicotine through β2-containing heteromeric nAChRs.

Nicotine-induced enhancement of excitatory activity was observed in brain slices from α7 nAChR knockout mice, but not in β2 knockout mice, demonstrating that the activation of α7- and β2-containing nAChRs differentially facilitates LTP induction via endogenously released ACh and exogenous nicotine, respectively [72]. A subsequent work from these authors studied the effects of nicotine and nicotinic antagonists on LTD induction in hippocampal slices prepared from α2, α7, and β2 subunit-containing knockout mice and wild-type animals [73]. LTD induction activates α7 nAChRs, and this activation generates a feedback mechanism whereby LTD induction is suppressed. In turn, nicotine application counteracts the feedback loop, resulting in even larger LTD mediated via non-α7 nAChR subtypes (Figure 4). The selective antagonist mecamylamine (but not dihydro-β-erythroidine) blocks the α7 nAChR-mediated effect, still observed in α2 or β2 knockout mice. Nakauchi and Sumikawa concluded that the loss of cholinergic projections to the hippocampus associated with diminished ACh release, as observed in Alzheimer patients, can affect LTD induction. Nicotine activates the PI3K/Akt and ERK/CREB pathways that increase the expression of the neurotrophic factor, BDNF. In addition, potentiation of synaptic efficacy is also observed through CaMKII activation [60].

## 5. Positive Allosteric Modulators (PAMs) and Silent Agonists of the nAChR

**Figure 5:**
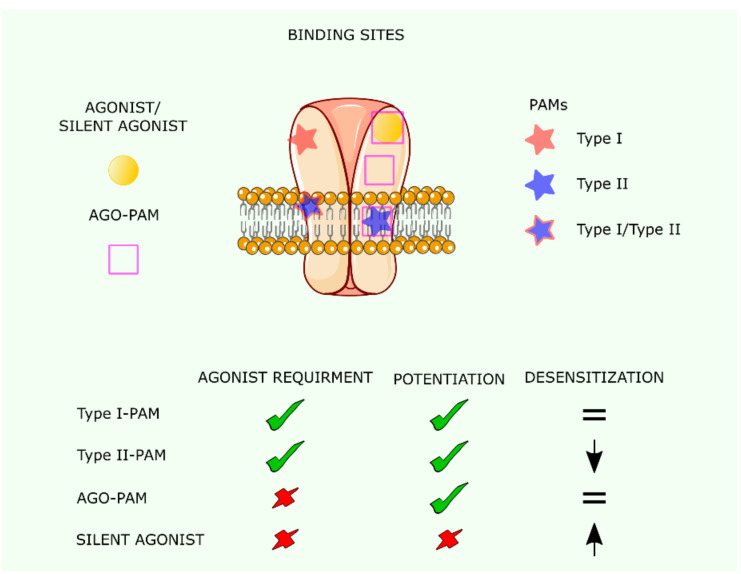
Pharmacology of α7 nAChR positive allosteric modulators (PAMs), agonist-PAMs, and silent agonists. The orthosteric binding sites are located at the interface of two subunits and are used by agonists, ago-PAM, and silent agonists. Allosteric sites were described in the transmembrane domain 2 (TM2) between the extracellular domain (ECD) and the TMD, at the ECD and in the interface between subunits in the ECD. PAMs and ago-PAMs use these sites to potentiate activation through the orthosteric site. The bottom part of the figure shows the effect of the different types of ligands on α7 nAChR-mediated currents.

nAChRs are allosteric proteins; not only does binding of the agonist to a binding site increase the affinity of the other site/s, but the binding of certain molecules at a locus different from the canonical (orthosteric) agonist-recognition site also synergizes the signal elicited by co-bound agonist. PAMs do not exert effects on nAChR unless an agonist is bound to the orthosteric site of the receptor, in which case the agonist-mediated activation is coupled with the PAM-mediated potentiation on the allosteric site (Figure 5). This is an advantage for the therapeutic application of PAMs, except for situations in which ACh biosynthesis is compromised, as is the case at advanced stages of Alzheimer disease (AD). Another advantage of PAMs as pharmacological agents is that they are more selective than agonists. The α7 nAChR is particularly sensitive to potentiation by PAMs [74,75]. 

Type I PAMs are compounds that increase α7 nAChR-mediated currents without affecting desensitization kinetics [76]. Binding sites for type I PAMs were described in the extracellular domain and the transmembrane region of the α7 nAChR [77] (Figure 5). In addition to potentiating orthosteric activation, type II PAMs decrease the rate of desensitization of the α7 nAChR [77]. In silico studies have shown an intra-subunit site to be important for type II PAM action [78]. In addition, mutations in the transmembrane domain of the five α7 nAChR subunits abolished the response of α7 nAChR to PNU-120596, a prototype type II PAM [79]. Interestingly, the currents evoked by PAMs are different from those evoked by agonists acting on the canonical orthosteric site. PAM-mediated currents do not show inward rectification and respond differently to antagonists such as mecamylamine [80,81]. The use of PAMs as therapeutic drugs has been discussed previously [82]. 

In recent years, a new type of α7 nAChR ligands termed ago-PAMs has been described (see Figure 5). These compounds not only potentiate orthosteric activation but can also directly activate the α7 nAChR in the same way as an agonist [83]. GAT107, B973B, and 4BP-TQS are examples of this class of molecules. GAT107 application to mice with induced experimental autoimmune encephalomyelitis (a model for multiple sclerosis) decreases the extent of neuroinflammation and reduces the severity of the disease [84]. In addition, dose-dependent antinociceptive effects of GAT107 in mouse models of acute and chronic pain were demonstrated. The effect was blocked by the specific antagonist mecamylamine and was not observed in α7 nAChR null mice, thus confirming that these therapeutic actions were mediated through α7 nAChRs [85]. 

Silent agonists, such as NS6740, are α7 nAChR-selective weak partial agonists, promoting the conversion of these receptors to a reversible desensitized state upon application of PAMs. In the case of the α7 nAChR, these non-conducting states were suggested to elicit signal transduction independently of ion channel opening. Indeed, although NS6740 does not activate α7 nAChR currents, it reduces lipopolysaccharide (LPS)-induced tumor necrosis factor α (TNF-α) release in microglia cells [86] and shows antinociceptive activity in animal models of inflammatory and neuropathic pain [87]. Agonists alone do not cause these beneficial effects, indicating that in these pathological conditions α7 nAChRs are able to signal independently of ion channel flux. In contrast, the silent agonist NS6740 reverses the enhancement in memory capacity induced by the partial agonist BMS-902483 in novel object recognition memory tests [88], prevents nicotine-induced LTP in the dentate gyrus, and reduces basal synaptic activity [89], indicating that these processes require ion influx through α7 nAChRs. 

Calcium permeability of α7 nAChRs is still a subject of intense investigation, particularly when discussing the possibility of using this receptor as a therapeutic target. Cytotoxicity leading to cell death was observed upon overactivation of α7 nAChRs in neuroblastoma cells treated with the PAM II drug PNU120596 [90]. ACh-evoked currents potentiated by GAT107 or B-973B showed reduced calcium permeability, although intracellular calcium was observed to rise [91]. Silent agonists can overcome this reduced calcium permeability under conditions that do not require ion flux for signaling [92].

## 6. Dendritic Spines and nAChR-Mediated Synaptic Plasticity 

α7 nAChRs are involved in dendritogenesis and in the maturation of glutamatergic synapses [93,94,95] and β2 subunit-containing nAChRs modulate the formation of dendritic spines and regulate dendritic morphology [96,97,98,99]. Plastic remodeling of dendritic spine morphology is observed upon application of nicotinic ligands that activate neuronal nAChRs and modulate the induction or inhibition of LTP and LTD, the functional correlates of learning and memory. Nicotine facilitates the induction of LTP in CA1 hippocampal neurons elicited by a weak tetanic stimulation that does not by itself produce LTP, while mecamylamine and the non-α7 nAChR antagonist dihydro-β-erythroidine inhibit LTP. The combination of nicotine and methyllycaconitine, an α7 nAChR antagonist, result in a form of LTD that can be blocked by dihydro-β-erythroidine. nAChR intervention in nicotine-facilitated synaptic plasticity involves the excitatory glutamatergic synapse [92]. The nicotine-facilitated LTP in lead-exposed rats is blocked by the NMDA-R antagonist d-(−)-2-amino-5-phosphonopentanoic acid, or picrotoxin, an antagonist of GABA_A_ receptors, leading Wang and coworkers to suggest that nicotine-facilitated synaptic plasticity is due to the activation of NMDARs by presynaptic nAChR-mediated disinhibition of pyramidal cells [100].

Nicotinic enhancement of α7 nAChR-mediated LTP in the dentate gyrus is not only NMDA-R-dependent but also requires the activation of metabotropic glutamate receptors (mGluRs) and Ca^2+^ influx via L-type Ca^2+^ channels and release from ryanodine-sensitive intracellular stores [101]. 

The nicotine/α7 nAChR-mediated LTP induction operates via acute and chronic mechanisms. Initiation of the acute nicotinic enhancement requires the activation of extracellular signal-regulated kinase (ERK), cAMP-dependent protein kinase (PKA), and phosphoinositide 3-kinase, whereas the chronic variant of nicotine enhancement of LTP depends on PKA, ERK, and Src kinases [102]. Peptide inhibitors of Src kinases inhibit tetanus-induced LTP. Conversely, in chronic nicotine-exposed cells, the inhibitor is ineffective in blocking tetanus-induced LTP [94].

Using a rodent model and based on the notion that α7 nAChR knockout mice present clear behavioral and mnemonic deficits, Morley and Mervis studied the morphology of dendrites in the CA1 region of the mouse hippocampus [94]. Golgi-stained sections from wildtype and α7 nAChR-KO mice at postnatal day 24 showed a 64% increase in what these authors call L (“lollipop” or thin spine neck) dendritic spines on the CA1 basilar tree of nAChR-KO mice, and small decreases in the number of “nubby” (N, or poorly defined spine head merged into a thickened spine neck)-type (−15%), mushroom (M)-type (−14%) and dimple (D, small with no distinct spine head)-type (−4%) dendritic spine densities. The dendritic arborization of knockout mice was significantly less developed close to the neuronal soma than in wild-type animals, but not in the more distal regions. The authors concluded that one component of cognitive dysfunction may be associated with α7 nAChR-modulated GABAergic interneurons synapsing on CA1 basal dendrites. Changes in CA1 basilar dendritic spines suggest that they are highly plastic. As mentioned before, one manifestation of this plastic behavior is the LTD-induced by the cholinergic system in hippocampus. 

Dendritic spine changes have been reported following nicotine-induced α4β2 nAChR activation on glutamatergic presynaptic termini [58]. The nicotine-induced lateral enlargement of spine heads was abolished by the α4β2 nAChR antagonist dihydro-β-erythroidine, but not by α-bungarotoxin, the canonical quasi-irreversible antagonist of α7 nAChRs. Tetanus toxin or antagonists of NMDA- and AMPA-type glutamate receptor blocked the nicotine-induced spine changes in morphology. Nicotine still produced full spine-enlarging responses in the postsynaptic neuron whose β2 nAChR expression was knocked down. These data suggest that nicotine influences the activity of glutamatergic neurotransmission through the activation of presynaptic α4β2 nAChRs, resulting in the modulation of spinal architecture and responsiveness. 

## 7. α7 nAChR in Disorders of the CNS

Due to its ubiquitous distribution and the multiple functions it displays in the brain, the α7 nAChR is associated with a variety of neurological and neuropsychiatric disorders [103,104,105,106,107,108,109,110,111]. α7 nAChR expression is reduced in the hippocampus, thalamus, and frontal cortex of patients with schizophrenia spectrum disorders [112,113,114,115]. Single nucleotide polymorphisms of the α7 nAChR CHRNA7 gene promoter are genetic markers linked with schizophrenia spectrum disorders [116,117]. Reduced expression of α7 nAChR has been associated with impaired auditory sensory gating, a hallmark of these disorders and related nosological entities. Sensory gating is the ability to filter out redundant stimuli, a mechanism required for good cognitive performance. An important diminution in the number of glutamatergic excitatory synapses has been observed in α7 nAChR-knockout mice [118]. Recently, the combined use of the α7 nAChR selective agonist CDP-choline and the cholinesterase inhibitor galantamine has been reported to improve this pathological condition [119]. 

Pharmacological intervention with cholinergic ligands has been experimentally assayed in models of depressive disorders. Chronic nicotine treatment rescues depressive-like behavior in CaMKIV null-mice, an effect that is mediated through α7 nAChRs, activation of the PI3K/Akt and ERK/CREB pathways, and augmented expression of BDNF. Galantamine is also a drug currently used to overcome the cholinergic deficit in Alzheimer disease. The link between the cholinergic system and Alzheimer disease is thought to occur through different mechanisms [120]. The cholinergic deficit in Alzheimer disease is well documented, as is the direct interaction between the β-amyloid peptide and the α7 nAChR [121,122]. Moreover, the result of Aβ-α7 nAChR interaction depends on Aβ concentration. At physiological concentrations (pM to low nM range), Aβ triggers α7 nAChR channel openings, while at higher concentrations (nM to low µM range), Aβ acts as a negative modulator [1]. Aβ induces changes in α7 nAChR conformation: a novel desensitized conformation still able to change in the presence of agonists was described at low Aβ concentrations [123]. 

Changes in spine size and density are observed at early stages in mouse models of Alzheimer disease [124]. Since spine morphology is largely mediated by the actin cytoskeletal network, the connection between Alzheimer disease markers and actin dynamics is an obvious subject of research. Indeed, the amyloid peptide Aβ1-42 has been shown to decrease actin dynamics in vitro and reduce the F-actin/G-actin ratio in synaptosomes of APPswe/PS1ΔE9 mice at as early as 1 month of age [125]. This effect precedes amyloid plaque formation and follows disease progression [126,127]. Aβ oligomers induce the activation of calcineurin, which results in actin depolymerization and subsequent spine dystrophy and eventually loss of actin fibers [128,129]. Interestingly, in mice models of Alzheimer disease, older animals show reduced spine density not only in the proximity of amyloid plaques but also at distant areas [130]. However, those spines close to the plaques (average distance ~6 µm) have higher F-actin content, suggesting that decreased actin dynamics is responsible for the reduced spine plasticity [131]. Deposition of Aβ in plaques would decrease the availability of soluble forms of Aβ, thus precluding signaling towards actin depolymerization in an attempt to protect spine structure. This mechanism may be exhausted at some point in the evolution of the disease and may explain the reduced levels of F-actin measured in synaptosomes from human postmortem brain tissue in Alzheimer patients with mild cognitive impairment [125,126].

Current studies on synaptopathies possibly linked to early stages of age-associated memory impairment (AAMI), considered one of the earliest cognitive functions to decline during aging, focus on other protein targets. In a transgenic murine model of aged brain transcription factor EB (TFEB), the master regulator of the autophagy–lysosome pathway that is differentially expressed in an age-dependent fashion in different brain regions, TFEB is reduced relative to control wild-type mice [61]. Importantly, this reduction significantly affects the frontal cortex and hippocampus, two brain regions involved in cognitive and mnemonic functions. Ectopic overexpression of transcription factor EB (TFEB) in transgenic mice mitigated the expression of senescence markers including P16INK4a, lamin B1, or H2A histone family member X (γ-H2AX) in the hippocampus and cerebral cortex, suggesting anti-aging effects and pointing to TFEB as a possible therapeutic target to ameliorate cognitive decline [132]. 

Cognitive resilience is defined as the ability to maintain normal cognition under brain disease conditions, including trauma, stroke, or the characteristic hallmarks of Alzheimer disease (neurofibrillary tangles and amyloid fiber deposits) [133]. Walker and coworkers discuss the hypothesis that dendritic spine plasticity is at the root of cognitive resilience to stress and, in particular, in the context of Alzheimer disease, where synaptic plasticity may be as important as genetic and lifestyle factors.

## 8. Conclusions

The homomeric α7 nAChR, with its five identical subunits, appears to be the simplest receptor within the nAChR family in its rapid ligand-gated homomeric version. It gains in complexity and versatility upon forming heteromeric oligomers with β subunits, whereby it can signal both ion fluxes and metabotropic pathways at different time scales. It is expressed in different cell types and different subcellular locations, thus engaging in multiple direct neurotransmitter and indirect modulatory roles in developing and mature tissues. These features make the α7 nAChR a central player in neurotransmission, Ca^2+^-mediated signals, and in the modulation of diverse fundamental processes in signal transduction, whereby its alteration is associated with several neurological and neuropsychiatric diseases. 

## References

[1] Alkondon M., Pereira E.F., Albuquerque E.X. (1998). α-Bungarotoxin-and Methyllycaconitine-Sensitive Nicotinic Receptors Mediate Fast Synaptic Transmission in Interneurons of Rat Hippocampal Slices. Brain Res..

[2] Yan Y., Peng C., Arvin M.C., Jin X.-T., Kim V.J., Ramsey M.D., Wang Y., Banala S., Wokosin D.L., McIntosh J.M. (2018). Nicotinic Cholinergic Receptors in VTA Glutamate Neurons Modulate Excitatory Transmission. Cell Rep..

[3] Hellström-Lindahl E., Gorbounova O., Seiger Å., Mousavi M., Nordberg A. (1998). Regional Distribution of Nicotinic Receptors during Prenatal Development of Human Brain and Spinal Cord. Dev. Brain Res..

[4] Murakami K., Ishikawa Y., Sato F. (2013). Localization of A7 Nicotinic Acetylcholine Receptor Immunoreactivity on GABAergic Interneurons in Layers I–III of the Rat Retrosplenial Granular Cortex. Neuroscience.

[5] Caruncho H.J., Guidotti A., Lindstrom J., Costa E., Pesold C. (1997). Subcellular Localization of the A7 Nicotinic Receptor in Rat Cerebellar Granule Cell Layer. Neuroreport.

[6] Zoli M., Pucci S., Vilella A., Gotti C. (2018). Neuronal and Extraneuronal Nicotinic Acetylcholine Receptors. Curr. Neuropharmacol..

[7] Picciotto M.R., Higley M.J., Mineur Y.S. (2012). Acetylcholine as a Neuromodulator: Cholinergic Signaling Shapes Nervous System Function and Behavior. Neuron.

[8] El-Bizri H., Clarke P.B.S. (1994). Blockade of Nicotinic Receptor-Mediated Release of Dopamine from Striatal Synaptosomes by Chlorisondamine Administered in Vivo. Br. J. Pharmacol..

[9] Lena C., Changeux J.-P., Mulle C. (1993). Evidence for “Preterminal” Nicotinic Receptors on GABAergic Axons in the Rat Interpeduncular Nucleus. J. Neurosci..

[10] Bali Z.K., Nagy L.V., Hernádi I. (2017). Alpha7 Nicotinic Acetylcholine Receptors Play a Predominant Role in the Cholinergic Potentiation of N-Methyl-D-Aspartate Evoked Firing Responses of Hippocampal CA1 Pyramidal Cells. Front. Cell. Neurosci..

[11] El-Bizri H., Clarke P.B.S. (1994). Blockade of Nicotinic Receptor-Mediated Release of Dopamine from Striatal Synaptosomes by Chlorisondamine and Other Nicotinic Antagonists Administered in Vitro. Br. J. Pharmacol..

[12] Koukouli F., Maskos U. (2015). The Multiple Roles of the A7 Nicotinic Acetylcholine Receptor in Modulating Glutamatergic Systems in the Normal and Diseased Nervous System. Biochem. Pharmacol..

[13] Rosenthal J.S., Yin J., Long C., Spillman E., Sheng C., Yuan Q. (2019). Temporal Regulation of Nicotinic Acetylcholine Receptor Subunits Supports Central Cholinergic Synapse Development. bioRxiv.

[14] Voytenko L.P., Lushnikova I.V., Savotchenko A.V., Isaeva E.V., Skok M.V., Lykhmus O.Y., Patseva M.A., Skibo G.G. (2015). Hippocampal GABAergic Interneurons Coexpressing Alpha7-Nicotinic Receptors and Connexin-36 Are Able to Improve Neuronal Viability under Oxygen–Glucose Deprivation. Brain Res..

[15] De Jaco A., Bernardini L., Rosati J., Maria Tata A. (2017). Alpha-7 Nicotinic Receptors in Nervous System Disorders: From Function to Therapeutic Perspectives. Cent. Nerv. Syst. Agents Med. Chem..

[16] Stojakovic A., Espinosa E.P., Farhad O.T., Lutfy K. (2017). Effects of Nicotine on Homeostatic and Hedonic Components of Food Intake. J. Endocrinol..

[17] Colangelo C., Shichkova P., Keller D., Markram H., Ramaswamy S. (2019). Cellular, Synaptic and Network Effects of Acetylcholine in the Neocortex. Front. Neural Circuits.

[18] Gandelman J.A., Newhouse P., Taylor W.D. (2018). Nicotine and Networks: Potential for Enhancement of Mood and Cognition in Late-Life Depression. Neurosci. Biobehav. Rev..

[19] Lykhmus O., Kalashnyk O., Uspenska K., Skok M. (2020). Positive Allosteric Modulation of Alpha7 Nicotinic Acetylcholine Receptors Transiently Improves Memory but Aggravates Inflammation in LPS-Treated Mice. Front. Aging Neurosci..

[20] Sabec M.H., Wonnacott S., Warburton E.C., Bashir Z.I. (2018). Nicotinic Acetylcholine Receptors Control Encoding and Retrieval of Associative Recognition Memory through Plasticity in the Medial Prefrontal Cortex. Cell Rep..

[21] Koukouli F., Rooy M., Changeux J.-P., Maskos U. (2016). Nicotinic Receptors in Mouse Prefrontal Cortex Modulate Ultraslow Fluctuations Related to Conscious Processing. Proc. Natl. Acad. Sci. USA.

[22] Caton M., Ochoa E.L., Barrantes F.J. (2020). The Role of Nicotinic Cholinergic Neurotransmission in Delusional Thinking. NPJ Schizophr..

[23] Pucci S., Fasoli F., Moretti M., Benfante R., Di Lascio S., Viani P., Daga A., Gordon T.J., McIntosh M., Zoli M. (2021). Choline and Nicotine Increase Glioblastoma Cell Proliferation by Binding and Activating A7-and A9-Containing Nicotinic Receptors. Pharmacol. Res..

[24] Zhao X., Wilson K., Uteshev V., He J.J. (2021). Activation of A7 Nicotinic Acetylcholine Receptor Ameliorates HIV-Associated Neurology and Neuropathology. Brain.

[25] Changeux J.-P. (2018). The Nicotinic Acetylcholine Receptor: A Typical ‘Allosteric Machine’. Philos. Trans. R. Soc. B Biol. Sci..

[26] Girod R., Crabtree G., Ernstrom G., Ramirez-Latorre J., McGehee D., Turner J., Role L. (1999). Heteromeric Complexes of A5 and/or A7 Subunits: Effects of Calcium and Potential Role in Nicotine-Induced Presynaptic Facilitation. Ann. N. Y. Acad. Sci..

[27] Khiroug S.S., Harkness P.C., Lamb P.W., Sudweeks S.N., Khiroug L., Millar N.S., Yakel J.L. (2002). Rat Nicotinic ACh Receptor A7 and Β2 Subunits Co-Assemble to Form Functional Heteromeric Nicotinic Receptor Channels. J. Physiol..

[28] Palma E., Maggi L., Barabino B., Eusebi F., Ballivet M. (1999). Nicotinic Acetylcholine Receptors Assembled from the A7 and Β3 Subunits. J. Biol. Chem..

[29] Criado M., Valor L.M., Mulet J., Gerber S., Sala S., Sala F. (2012). Expression and Functional Properties of A7 Acetylcholine Nicotinic Receptors Are Modified in the Presence of Other Receptor Subunits. J. Neurochem..

[30] Cui C., Booker T.K., Allen R.S., Grady S.R., Whiteaker P., Marks M.J., Salminen O., Tritto T., Butt C.M., Allen W.R. (2003). The Β3 Nicotinic Receptor Subunit: A Component of α-Conotoxin MII-Binding Nicotinic Acetylcholine Receptors That Modulate Dopamine Release and Related Behaviors. J. Neurosci..

[31] Drisdel R.C., Green W.N. (2000). Neuronal α-Bungarotoxin Receptors Are A7 Subunit Homomers. J. Neurosci..

[32] Gotti C., Hanke W., Maury K., Moretti M., Ballivet M., Clementi F., Bertrand D. (1994). Pharmacology and Biophysical Properties of A7 and A7-A8 α-Bungarotoxin Receptor Subtypes Immunopurified from the Chick Optic Lobe. Eur. J. Neurosci..

[33] Liu Q., Huang Y., Xue F., Simard A., DeChon J., Li G., Zhang J., Lucero L., Wang M., Sierks M. (2009). A Novel Nicotinic Acetylcholine Receptor Subtype in Basal Forebrain Cholinergic Neurons with High Sensitivity to Amyloid Peptides. J. Neurosci..

[34] Dajas-Bailador F.A., Mogg A.J., Wonnacott S. (2002). Intracellular Ca^2+^ Signals Evoked by Stimulation of Nicotinic Acetylcholine Receptors in SH-SY5Y Cells: Contribution of Voltage-Operated Ca^2+^ Channels and Ca^2+^ Stores. J. Neurochem..

[35] Kabbani N., Nichols R.A. (2018). Beyond the Channel: Metabotropic Signaling by Nicotinic Receptors. Trends Pharmacol. Sci..

[36] Moretti M., Zoli M., George A.A., Lukas R.J., Pistillo F., Maskos U., Whiteaker P., Gotti C. (2014). The Novel A7β2-Nicotinic Acetylcholine Receptor Subtype Is Expressed in Mouse and Human Basal Forebrain: Biochemical and Pharmacological Characterization. Mol. Pharmacol..

[37] King J.R., Kabbani N. (2016). Alpha 7 Nicotinic Receptor Coupling to Heterotrimeric G Proteins Modulates RhoA Activation, Cytoskeletal Motility, and Structural Growth. J. Neurochem..

[38] King J.R., Nordman J.C., Bridges S.P., Lin M.-K., Kabbani N. (2015). Identification and Characterization of a G Protein-Binding Cluster in A7 Nicotinic Acetylcholine Receptors. J. Biol. Chem..

[39] Thomsen M.S., Zwart R., Ursu D., Jensen M.M., Pinborg L.H., Gilmour G., Wu J., Sher E., Mikkelsen J.D. (2015). α7 and β2 Nicotinic Acetylcholine Receptor Subunits Form Heteromeric Receptor Complexes that Are Expressed in the Human Cortex and Display Distinct Pharmacological Properties. PLoS ONE.

[40] Nielsen B.E., Minguez T., Bermudez I., Bouzat C. (2018). Molecular function of the novel α7β2 nicotinic receptor. Cell Mol. Life Sci..

[41] George A.A., Vieira J.M., Xavier-Jackson C., Gee M.T., Cirrito J.R., Bimonte-Nelson H.A., Picciotto M.R., Lukas R.J., Whiteaker P. (2021). Implications of Oligomeric Amyloid-Beta (oAβ42) Signaling through α7β2-Nicotinic Acetylcholine Receptors (nAChRs) on Basal Forebrain Cholinergic Neuronal Intrinsic Excitability and Cognitive Decline. J. Neurosci..

[42] Donat C.K., Hansen H.H., Hansen H.D., Mease R.C., Horti A.G., Pomper M.G., L’Estrade E.T., Herth M.M., Peters D., Knudsen G.M. (2020). In Vitro and In Vivo Characterization of Dibenzothiophene Derivatives [^125^I]Iodo-ASEM and [18F]ASEM as Radiotracers of Homo- and Heteromeric α7 Nicotinic Acetylcholine Receptors. Molecules.

[43] Richter K., Mathes V., Fronius M., Althaus M., Hecker A., Krasteva-Christ G., Padberg W., Hone A.J., McIntosh J.M., Zakrzewicz A. (2016). Phosphocholine—An agonist of metabotropic but not of ionotropic functions of α9-containing nicotinic acetylcholine receptors. Sci. Rep..

[44] Sarter M., Parikh V. (2005). Choline Transporters, Cholinergic Transmission and Cognition. Nat. Rev. Neurosci..

[45] Hecker A., Küllmar M., Wilker S., Richter K., Zakrzewicz A., Atanasova S., Mathes V., Timm T., Lerner S., Klein J. (2015). Phosphocholine-Modified Macromolecules and Canonical Nicotinic Agonists Inhibit ATP-Induced IL-1β Release. J. Immunol..

[46] Papke R.L., Bencherif M., Lippiello P. (1996). An Evaluation of Neuronal Nicotinic Acetylcholine Receptor Activation by Quaternary Nitrogen Compounds Indicates That Choline Is Selective for the A7 Subtype. Neurosci. Lett..

[47] Parikh V., Pomerleau F., Huettl P., Gerhardt G.A., Sarter M., Bruno J.P. (2004). Rapid Assessment of in Vivo Cholinergic Transmission by Amperometric Detection of Changes in Extracellular Choline Levels. Eur. J. Neurosci..

[48] Mucchietto V., Fasoli F., Pucci S., Moretti M., Benfante R., Maroli A., Di Lascio S., Bolchi C., Pallavicini M., Dowell C. (2018). A9-and A7-Containing Receptors Mediate the pro-Proliferative Effects of Nicotine in the A549 Adenocarcinoma Cell Line. Br. J. Pharmacol..

[49] Bavo F., Pucci S., Fasoli F., Lammi C., Moretti M., Mucchietto V., Lattuada D., Viani P., De Palma C., Budriesi R. (2018). Potent Antiglioblastoma Agents by Hybridizing the Onium-Alkyloxy-Stilbene Based Structures of an A7-NAChR, A9-NAChR Antagonist and of a pro-Oxidant Mitocan. J. Med. Chem..

[50] De Robertis E.D. (2013). Histophysiology of Synapses and Neurosecretion: International Series of Monographs on Pure and Applied Biology: Modern Trends in Physiological Sciences.

[51] Südhof T.C. (2021). The Cell Biology of Synapse Formation. J. Cell Biol..

[52] Fabian-Fine R., Skehel P., Errington M.L., Davies H.A., Sher E., Stewart M.G., Fine A. (2001). Ultrastructural Distribution of the A7 Nicotinic Acetylcholine Receptor Subunit in Rat Hippocampus. J. Neurosci..

[53] Hazan L., Ziv N.E. (2020). Activity Dependent and Independent Determinants of Synaptic Size Diversity. J. Neurosci..

[54] Lacor P.N., Buniel M.C., Furlow P.W., Clemente A.S., Velasco P.T., Wood M., Viola K.L., Klein W.L. (2007). Aβ Oligomer-Induced Aberrations in Synapse Composition, Shape, and Density Provide a Molecular Basis for Loss of Connectivity in Alzheimer’s Disease. J. Neurosci..

[55] Bourne J., Harris K.M. (2007). Do Thin Spines Learn to Be Mushroom Spines That Remember?. Curr. Opin. Neurobiol..

[56] Kasai H., Ziv N.E., Okazaki H., Yagishita S., Toyoizumi T. (2021). Spine Dynamics in the Brain, Mental Disorders and Artificial Neural Networks. Nat. Rev. Neurosci..

[57] Ziv N.E., Brenner N. (2018). Synaptic Tenacity or Lack Thereof: Spontaneous Remodeling of Synapses. Trends Neurosci..

[58] Oda A., Yamagata K., Nakagomi S., Uejima H., Wiriyasermkul P., Ohgaki R., Nagamori S., Kanai Y., Tanaka H. (2014). Nicotine Induces Dendritic Spine Remodeling in Cultured Hippocampal Neurons. J. Neurochem..

[59] Galvez B., Gross N., Sumikawa K. (2016). Activation of A7 Nicotinic Acetylcholine Receptors Protects Potentiated Synapses from Depotentiation during Theta Pattern Stimulation in the Hippocampal CA1 Region of Rats. Neuropharmacology.

[60] Moriguchi S., Inagaki R., Yi L., Shibata M., Sakagami H., Fukunaga K. (2020). Nicotine Rescues Depressive-like Behaviors via A7-Type Nicotinic Acetylcholine Receptor Activation in CaMKIV Null Mice. Mol. Neurobiol..

[61] Shen J., Yakel J.L. (2009). Nicotinic Acetylcholine Receptor-Mediated Calcium Signaling in the Nervous System. Acta Pharmacol. Sin..

[62] Unwin N. (2013). Nicotinic Acetylcholine Receptor and the Structural Basis of Neuromuscular Transmission: Insights from Torpedo Postsynaptic Membranes. Q. Rev. Biophys..

[63] Fasoli F., Gotti C. (2015). Structure of Neuronal Nicotinic Receptors. Neurobiol. Genet. Nicotine Tob..

[64] Morales-Perez C.L., Noviello C.M., Hibbs R.E. (2016). X-Ray Structure of the Human A4β2 Nicotinic Receptor. Nature.

[65] Noviello C.M., Gharpure A., Mukhtasimova N., Cabuco R., Baxter L., Borek D., Sine S.M., Hibbs R.E. (2021). Structure and Gating Mechanism of the A7 Nicotinic Acetylcholine Receptor. Cell.

[66] Walsh R.M., Roh S.-H., Gharpure A., Morales-Perez C.L., Teng J., Hibbs R.E. (2018). Structural Principles of Distinct Assemblies of the Human A4β2 Nicotinic Receptor. Nature.

[67] Son C.D., Moss F.J., Cohen B.N., Lester H.A. (2009). Nicotine Normalizes Intracellular Subunit Stoichiometry of Nicotinic Receptors Carrying Mutations Linked to Autosomal Dominant Nocturnal Frontal Lobe Epilepsy. Mol. Pharmacol..

[68] Weltzin M.M., Lindstrom J.M., Lukas R.J., Whiteaker P. (2016). Distinctive Effects of Nicotinic Receptor Intracellular-Loop Mutations Associated with Nocturnal Frontal Lobe Epilepsy. Neuropharmacology.

[69] Kouvatsos N., Giastas P., Chroni-Tzartou D., Poulopoulou C., Tzartos S.J. (2016). Crystal structure of a human neuronal nAChR extracellular domain in pentameric assembly: Ligand-bound α2 homopentamer. Proc. Natl. Acad. Sci. USA.

[70] Andersen N., Corradi J., Sine S.M., Bouzat C. (2013). Stoichiometry for Activation of Neuronal α7 Nicotinic Receptors. Proc. Natl. Acad. Sci. USA.

[71] Bartsch T., Döhring J., Rohr A., Jansen O., Deuschl G. (2011). CA1 Neurons in the Human Hippocampus Are Critical for Autobiographical Memory, Mental Time Travel, and Autonoetic Consciousness. Proc. Natl. Acad. Sci. USA.

[72] Nakauchi S., Sumikawa K. (2012). Endogenously Released ACh and Exogenous Nicotine Differentially Facilitate Long-Term Potentiation Induction in the Hippocampal CA1 Region of Mice. Eur. J. Neurosci..

[73] Nakauchi S., Sumikawa K. (2014). Endogenous ACh Suppresses LTD Induction and Nicotine Relieves the Suppression via Different Nicotinic ACh Receptor Subtypes in the Mouse Hippocampus. Life Sci..

[74] Horenstein N.A., Papke R.L., Kulkarni A.R., Chaturbhuj G.U., Stokes C., Manther K., Thakur G.A. (2016). Critical Molecular Determinants of A7 Nicotinic Acetylcholine Receptor Allosteric Activation: Separation of Direct Allosteric Activation and Positive Allosteric Modulation. J. Biol. Chem..

[75] Papke R.L., Lindstrom J.M. (2020). Nicotinic Acetylcholine Receptors: Conventional and Unconventional Ligands and Signaling. Neuropharmacology.

[76] Grønlien J.H., Håkerud M., Ween H., Thorin-Hagene K., Briggs C.A., Gopalakrishnan M., Malysz J. (2007). Distinct Profiles of A7 NAChR Positive Allosteric Modulation Revealed by Structurally Diverse Chemotypes. Mol. Pharmacol..

[77] Targowska-Duda K.M., Kaczor A.A., Jozwiak K., Arias H.R. (2019). Molecular Interactions of Type I and Type II Positive Allosteric Modulators with the Human A7 Nicotinic Acetylcholine Receptor: An in silico Study. J. Biomol. Struct. Dyn..

[78] Corrie J.B., Free C.R., Corradi J., Bouzat C., Sine S.M. (2011). Single-Channel and Structural Foundations of Neuronal A7 Acetylcholine Receptor Potentiation. J. Neurosci..

[79] Sitzia F., Brown J.T., Randall A., Dunlop J. (2011). Voltage-and Temperature-Dependent Allosteric Modulation of A7 Nicotinic Receptors by PNU120596. Front. Pharmacol..

[80] Peng C., Kimbrell M.R., Tian C., Pack T.F., Crooks P.A., Fifer E.K., Papke R.L. (2013). Multiple Modes of A7 NAChR Noncompetitive Antagonism of Control Agonist-Evoked and Allosterically Enhanced Currents. Mol. Pharmacol..

[81] Quadri M., Garai S., Thakur G.A., Stokes C., Gulsevin A., Horenstein N.A., Papke R.L. (2019). Macroscopic and Microscopic Activation of A7 Nicotinic Acetylcholine Receptors by the Structurally Unrelated Allosteric Agonist-Positive Allosteric Modulators (Ago-PAMs) B-973B and GAT107. Mol. Pharmacol..

[82] Vallés A.S., Borroni M.V., Barrantes F.J. (2014). Targeting Brain A7 Nicotinic Acetylcholine Receptors in Alzheimer’s Disease: Rationale and Current Status. CNS Drugs.

[83] Gill J.K., Savolainen M., Young G.T., Zwart R., Sher E., Millar N.S. (2011). Agonist Activation of A7 Nicotinic Acetylcholine Receptors via an Allosteric Transmembrane Site. Proc. Natl. Acad. Sci. USA.

[84] Mizrachi T., Marsha O., Brusin K., Ben-David Y., Thakur G.A., Vaknin-Dembinsky A., Treinin M., Brenner T. (2021). Suppression of Neuroinflammation by an Allosteric Agonist and Positive Allosteric Modulator of the A7 Nicotinic Acetylcholine Receptor GAT107. J. Neuroinflamm..

[85] Bagdas D., Wilkerson J.L., Kulkarni A., Toma W., AlSharari S., Gul Z., Lichtman A.H., Papke R.L., Thakur G.A., Damaj M.I. (2016). The A7 Nicotinic Receptor Dual Allosteric Agonist and Positive Allosteric Modulator GAT107 Reverses Nociception in Mouse Models of Inflammatory and Neuropathic Pain. Br. J. Pharmacol..

[86] Thomsen M.S., Mikkelsen J.D. (2012). The A7 Nicotinic Acetylcholine Receptor Ligands Methyllycaconitine, NS6740 and GTS-21 Reduce Lipopolysaccharide-Induced TNF-α Release from Microglia. J. Neuroimmunol..

[87] Papke R.L., Bagdas D., Kulkarni A.R., Gould T., AlSharari S.D., Thakur G.A., Damaj M.I. (2015). The Analgesic-like Properties of the Alpha7 NAChR Silent Agonist NS6740 Is Associated with Non-Conducting Conformations of the Receptor. Neuropharmacology.

[88] Pieschl R.L., Miller R., Jones K.M., Post-Munson D.J., Chen P., Newberry K., Benitex Y., Molski T., Morgan D., McDonald I.M. (2017). Effects of BMS-902483, an A7 Nicotinic Acetylcholine Receptor Partial Agonist, on Cognition and Sensory Gating in Relation to Receptor Occupancy in Rodents. Eur. J. Pharmacol..

[89] Papke R.L., Peng C., Kumar A., Stokes C. (2018). NS6740, an A7 Nicotinic Acetylcholine Receptor Silent Agonist, Disrupts Hippocampal Synaptic Plasticity. Neurosci. Lett..

[90] Guerra-Álvarez M., Moreno-Ortega A.J., Navarro E., Fernández-Morales J.C., Egea J., López M.G., Cano-Abad M.F. (2015). Positive Allosteric Modulation of Alpha-7 Nicotinic Receptors Promotes Cell Death by Inducing Ca^2+^ Release from the Endoplasmic Reticulum. J. Neurochem..

[91] Miller D.R., Khoshbouei H., Garai S., Cantwell L.N., Stokes C., Thakur G., Papke R.L. (2020). Allosterically Potentiated A7 Nicotinic Acetylcholine Receptors: Reduced Calcium Permeability and Current-Independent Control of Intracellular Calcium. Mol. Pharmacol..

[92] Pismataro M.C., Horenstein N.A., Stokes C., Quadri M., De Amici M., Papke R.L., Dallanoce C. (2020). Design, Synthesis, and Electrophysiological Evaluation of NS6740 Derivatives: Exploration of the Structure-Activity Relationship for Alpha7 Nicotinic Acetylcholine Receptor Silent Activation. Eur. J. Med. Chem..

[93] Campbell N.R., Fernandes C.C., Halff A.W., Berg D.K. (2010). Endogenous Signaling through A7-Containing Nicotinic Receptors Promotes Maturation and Integration of Adult-Born Neurons in the Hippocampus. J. Neurosci..

[94] Morley B.J., Mervis R.F. (2013). Dendritic Spine Alterations in the Hippocampus and Parietal Cortex of Alpha7 Nicotinic Acetylcholine Receptor Knockout Mice. Neuroscience.

[95] Lin H., Hsu F.C., Baumann B.H., Coulter D.A., Lynch D.R. (2014). Cortical Synaptic NMDA Receptor Deficits in A7 Nicotinic Acetylcholine Receptor Gene Deletion Models: Implications for Neuropsychiatric Diseases. Neurobiol. Dis..

[96] Ballesteros-Yáñez I., Benavides-Piccione R., Bourgeois J.P., Changeux J.P., DeFelipe J. (2010). Alterations of Cortical Pyramidal Neurons in Mice Lacking High-Affinity Nicotinic Receptors. Proc. Natl. Acad. Sci. USA.

[97] Lozada A.F., Wang X., Gounko N.V., Massey K.A., Duan J., Liu Z., Berg D.K. (2012). Induction of Dendritic Spines by Β2-Containing Nicotinic Receptors. J. Neurosci..

[98] Bailey C.D.C., Alves N.C., Nashmi R., De Biasi M., Lambe E.K. (2012). Nicotinic A5 Subunits Drive Developmental Changes in the Activation and Morphology of Prefrontal Cortex Layer VI Neurons. Biol. Psychiatry.

[99] Kang L., Tian M.K., Bailey C.D.C., Lambe E.K. (2015). Dendritic Spine Density of Prefrontal Layer 6 Pyramidal Neurons in Relation to Apical Dendrite Sculpting by Nicotinic Acetylcholine Receptors. Front. Cell. Neurosci..

[100] Wang H.-L., Chen X.-T., Luo L., Lou Z.-Y., Wang S., Chen J.-T., Wang M., Sun L.-G., Ruan D.-Y. (2006). Reparatory Effects of Nicotine on NMDA Receptor-Mediated Synaptic Plasticity in the Hippocampal CA1 Region of Chronically Lead-Exposed Rats. Eur. J. Neurosci..

[101] Welsby P., Rowan M., Anwyl R. (2006). Nicotinic Receptor-Mediated Enhancement of Long-Term Potentiation Involves Activation of Metabotropic Glutamate Receptors and Ryanodine-Sensitive Calcium Stores in the Dentate Gyrus. Eur. J. Neurosci..

[102] Welsby P.J., Rowan M.J., Anwyl R. (2009). Intracellular Mechanisms Underlying the Nicotinic Enhancement of LTP in the Rat Dentate Gyrus. Eur. J. Neurosci..

[103] Nordberg A. (1994). Human Nicotinic Receptors—Their Role in Aging and Dementia. Neurochem. Int..

[104] Nordberg A., Alafuzoff I., Winblad B. (1992). Nicotinic and Muscarinic Subtypes in the Human Brain: Changes with Aging and Dementia. J. Neurosci. Res..

[105] Leonard S., Adams C., Breese C.R., Adler L.E., Bickford P., Byerley W., Coon H., Griffith J.M., Miller C., Myles-Worsley M. (1996). Nicotinic Receptor Function in Schizophrenia. Schizophr. Bull..

[106] Leonard S., Gault J., Adams C., Breese C.R., Rollins Y., Adler L.E., Olincy A., Freedman R. (1998). Nicotinic Receptors, Smoking and Schizophrenia. Restor. Neurol. Neurosci..

[107] Mexal S., Berger R., Logel J., Ross R.G., Freedman R., Leonard S. (2010). Differential Regulation of A7 Nicotinic Receptor Gene (CHRNA7) Expression in Schizophrenic Smokers. J. Mol. Neurosci..

[108] Lewis A.S., Picciotto M.R. (2013). High-Affinity Nicotinic Acetylcholine Receptor Expression and Trafficking Abnormalities in Psychiatric Illness. Psychopharmacology.

[109] Weiland S., Bertrand D., Leonard S. (2000). Neuronal Nicotinic Acetylcholine Receptors: From the Gene to the Disease. Behav. Brain Res..

[110] Palma E., Conti L., Roseti C., Limatola C. (2012). Novel Approaches to Study the Involvement of A7-NAChR in Human Diseases. Curr. Drug Targets.

[111] Barrantes F.J. (1998). Molecular pathology of the nicotinic acetylcholine receptor. The Nicotinic Acetylcholine Receptor.

[112] Freedman R., Hall M., Adler L.E., Leonard S. (1995). Evidence in Postmortem Brain Tissue for Decreased Numbers of Hippocampal Nicotinic Receptors in Schizophrenia. Biol. Psychiatry.

[113] Olincy A., Stevens K.E. (2007). Treating Schizophrenia Symptoms with an A7 Nicotinic Agonist, from Mice to Men. Biochem. Pharmacol..

[114] Guan Z.-Z., Zhang X., Blennow K., Nordberg A. (1999). Decreased Protein Level of Nicotinic Receptor A7 Subunit in the Frontal Cortex from Schizophrenic Brain. Neuroreport.

[115] Court J., Spurden D., Lloyd S., McKeith I., Ballard C., Cairns N., Kerwin R., Perry R., Perry E. (1999). Neuronal Nicotinic Receptors in Dementia with Lewy Bodies and Schizophrenia: α-Bungarotoxin and Nicotine Binding in the Thalamus. J. Neurochem..

[116] Bertelsen B., Oranje B., Melchior L., Fagerlund B., Werge T.M., Mikkelsen J.D., Tümer Z., Glenthøj B.Y. (2015). Association Study of CHRNA7 Promoter Variants with Sensory and Sensorimotor Gating in Schizophrenia Patients and Healthy Controls: A Danish Case–Control Study. Neuromol. Med..

[117] Sinkus M.L., Graw S., Freedman R., Ross R.G., Lester H.A., Leonard S. (2015). The Human CHRNA7 and CHRFAM7A Genes: A Review of the Genetics, Regulation, and Function. Neuropharmacology.

[118] Lozada A.F., Wang X., Gounko N.V., Massey K.A., Duan J., Liu Z., Berg D.K. (2012). Glutamatergic Synapse Formation Is Promoted by A7-Containing Nicotinic Acetylcholine Receptors. J. Neurosci..

[119] Choueiry J., Blais C.M., Shah D., Smith D., Fisher D., Illivitsky V., Knott V. (2019). Combining CDP-Choline and Galantamine: Effects of a Selective A7 Nicotinic Acetylcholine Receptor Agonist Strategy on P50 Sensory Gating of Speech Sounds in Healthy Volunteers. J. Psychopharmacol..

[120] Oda A., Tanaka H. (2014). Activities of Nicotinic Acetylcholine Receptors Modulate Neurotransmission and Synaptic Architecture. Neural Regen. Res..

[121] Dineley K.T., Pandya A.A., Yakel J.L. (2015). Nicotinic ACh Receptors as Therapeutic Targets in CNS Disorders. Trends Pharmacol. Sci..

[122] Guan Z.-Z., Zhang X., Ravid R., Nordberg A. (2000). Decreased Protein Levels of Nicotinic Receptor Subunits in the Hippocampus and Temporal Cortex of Patients with Alzheimer’s Disease. J. Neurochem..

[123] Spires-Jones T.L., Meyer-Luehmann M., Osetek J.D., Jones P.B., Stern E.A., Bacskai B.J., Hyman B.T. (2007). Impaired Spine Stability Underlies Plaque-Related Spine Loss in an Alzheimer’s Disease Mouse Model. Am. J. Pathol..

[124] Lasala M., Fabiani C., Corradi J., Antollini S., Bouzat C. (2019). Molecular Modulation of Human A7 Nicotinic Receptor by Amyloid-β Peptides. Front. Cell. Neurosci..

[125] Kommaddi R.P., Das D., Karunakaran S., Nanguneri S., Bapat D., Ray A., Shaw E., Bennett D.A., Nair D., Ravindranath V. (2018). Aβ Mediates F-Actin Disassembly in Dendritic Spines Leading to Cognitive Deficits in Alzheimer’s Disease. J. Neurosci..

[126] Hrynchak M.V., Rierola M., Golovyashkina N., Penazzi L., Pump W.C., David B., Sündermann F., Brandt R., Bakota L. (2020). Chronic Presence of Oligomeric Aβ Differentially Modulates Spine Parameters in the Hippocampus and Cortex of Mice with Low APP Transgene Expression. Front. Synaptic Neurosci..

[127] Reese L.C., Zhang W., Dineley K.T., Kayed R., Taglialatela G. (2008). Selective Induction of Calcineurin Activity and Signaling by Oligomeric Amyloid Beta. Aging Cell.

[128] Stallings N.R., O’Neal M.A., Hu J., Kavalali E.T., Bezprozvanny I., Malter J.S. (2018). Pin1 Mediates Aβ42-Induced Dendritic Spine Loss. Sci. Signal..

[129] Wu H.-Y., Hudry E., Hashimoto T., Kuchibhotla K., Rozkalne A., Fan Z., Spires-Jones T., Xie H., Arbel-Ornath M., Grosskreutz C.L. (2010). Amyloid β Induces the Morphological Neurodegenerative Triad of Spine Loss, Dendritic Simplification, and Neuritic Dystrophies through Calcineurin Activation. J. Neurosci..

[130] Bittner T., Fuhrmann M., Burgold S., Ochs S.M., Hoffmann N., Mitteregger G., Kretzschmar H., LaFerla F.M., Herms J. (2010). Multiple Events Lead to Dendritic Spine Loss in Triple Transgenic Alzheimer’s Disease Mice. PLoS ONE.

[131] Kirkwood C.M., Ciuchta J., Ikonomovic M.D., Fish K.N., Abrahamson E.E., Murray P.S., Klunk W.E., Sweet R.A. (2013). Dendritic Spine Density, Morphology, and Fibrillar Actin Content Surrounding Amyloid-β Plaques in a Mouse Model of Amyloid-β Deposition. J. Neuropathol. Exp. Neurol..

[132] Wang H., Karuppan M.K.M., Devadoss D., Nair M., Chand H.S., Lakshmana M.K. (2021). TFEB protein expression is reduced in aged brains and its overexpression mitigates senescence-associated biomarkers and memory deficits in mice. Neurobiol. Aging.

[133] Walker C.K., Herskowitz J.H. (2020). Dendritic Spines: Mediators of Cognitive Resilience in Aging and Alzheimer’s Disease. Neuroscientist.

